# Effects of dexmedetomidine on cardiac electrophysiology in patients undergoing general anesthesia during perioperative period: a randomized controlled trial

**DOI:** 10.1186/s12871-022-01811-5

**Published:** 2022-08-25

**Authors:** Chao Tan, Shiting Yan, Jie Shen, Hao Wu, Leyang Yu, Ying Wang, Shunping Tian, Wei Zhou, Yong Wu, Zhuan Zhang

**Affiliations:** 1 Department of Anesthesiology, the Affiliated Hospital of Yangzhou University, Yangzhou, 225012 China; 2 Graduate School, Dalian Medical University, Dalian, 116000 China; 3 Department of Cardiac Function, the Affiliated Hospital of Yangzhou University, Yangzhou, 225012 China

**Keywords:** Dexmedetomidine, General anesthesia, Cardiac electrophysiology, Cardiac function

## Abstract

**Background:**

Dexmedetomidine has controversial influence on cardiac electrophysiology. The aim of this study was to explore the effects of dexmedetomidine on perioperative cardiac electrophysiology in patients undergoing general anesthesia.

**Methods:**

Eighty-one patients were randomly divided into four groups: groups D_1_, D_2_, D_3_ receiving dexmedetomidine 1, 1, 0.5 μg/kg over 10 min and 1, 0.5, 0.5 μg/kg/h continuous infusion respectively, and control group (group C) receiving normal saline. Twelve-lead electrocardiograms were recorded at the time before dexmedetomidine/normal saline infusion (T_1_), loading dose finish (T_2_), surgery ending (T_6_), 1 h (T_7_) after entering PACU, 24 h (T_8_), 48 h (T_9_), 72 h (T_10_) and 1 month (T_11_) postoperatively. Cardiac circulation efficiency (CCE) were also recorded.

**Results:**

Compared with group C, QTc were significantly increased at T_2_ in groups D_1_ and D_2_ while decreased at T_7_ and T_8_ in group D_3_ (*P* < 0.05), iCEB were decreased at T_8_ (*P* < 0.05). Compared with group D_1_, QTc at T_2_, T_6_, T_7_, T_9_ and T_10_ and iCEB at T_8_ were decreased, and CCE at T_2_-T_4_ were increased in group D_3_ significantly (*P* < 0.05). Compared with group D_2_, QTc at T_2_ and iCEB at T_8_ were decreased and CCE at T_2_ and T_3_ were increased in group D_3_ significantly (*P* < 0.05).

**Conclusions:**

Dexmedetomidine at a loading dose of 0.5 μg/kg and a maintenance dose of 0.5 μg/kg/h can maintain stability of cardiac electrophysiology during perioperative period and has no significant adverse effects on CCE.

**Trial registration:**

ClinicalTrials.gov NCT04577430 (Date of registration: 06/10/2020).

## Background

Dexmedetomidine is a highly selective α_2_-adrenergic receptor agonist, which can inhibit the activity of sympathetic nervous system and exert predictable sedative and analgesic effects without obvious respiratory depression [1, 2]. Previous studies [3–6] reported that dexmedetomidine could change the permeability of ion channels to affect cardiac electrophysiology balance and cardiac function during perioperative period. A previous study discovered that dexmedetomidine could intensify the efferent impulse of vagus nerve, prolong the effective refractory period of myocardial cells, decrease myocardial autonomy, and shorten the abnormally prolonged QTc [7]. While researchers found that dexmedetomidine could also prolong the QTc [8], inhibit the function of sinus and atrioventricular (AV) node in children [9, 10], and even induce refractory cardiogenic shock [6]. The commonly used anesthetics, propofol and remifentanil, for general anesthesia have little significant impacts on cardiac electrophysiology [11–17].

In the current study, dexmedetomidine was applied to patients undergoing elective surgeries for 1–3 h under total intravenous general anesthesia with continuous infusion of propofol and remifentanil. The first aim was to observe its influences on cardiac electrophysiology and function perioperatively. The secondary aim was to probe into the optimum dosage of dexmedetomidine that had minimum negative effects on electrocardia action and cardiac function during perioperative period.

## Material and methods

### Study design and approval

This prospective, double-blind, randomized controlled study was abided by the Consolidated Standards of Reporting Trials (CONSORT) regulations and conducted after gaining approval from the ethics committee of the Affiliated Hospital of Yangzhou University (2020-YKL09-025, China). Written informed consent following the principles of Declaration of Helsinki was obtained from all the subjects included in the study. The trial was registered before patient enrollments at clinicaltrials.gov (NCT04577430).

Totally 81 gender-neutral patients undergoing elective surgeries for 1–3 h under total intravenous anesthesia, aged 18–65 years of age, body mass index (BMI) 18.5–30.0 kg/m^2^, American Society of Anesthesiologists (ASA) status I or II, were eligible for study recruitment regulations. Exclusion criteria were as follows: refusal; hypersensitivity to study medication; diabetes; preoperative QTc prolongation (male ≥ 440 ms, female ≥ 460 ms); abnormal cardiac conduction and other arrhythmia; history of heart disease, such as pacemaker implantation, coronary heart disease, congestive heart failure, and heart valve disease, etc.; use of antiarrhythmic drugs that affect the QT interval, such as β-receptor blockers and calcium channel blockers, etc.; non-sinus rhythm, severe sinus bradycardia [Heart rate (HR) ≤ 50 beats/min]; electrolyte abnormalities before surgery; liver and kidney function abnormalities; or surgical procedures with cardiovascular, malignant, thoracic and other procedures lasting longer than 3 h.

The patients were assigned to four groups using a computer-generated number table randomly: dexmedetomidine loaded with 1 μg/kg and maintained with 1 μg/kg/h (group D_1_), 1 μg/kg and 0.5 μg/kg/h (group D_2_), 0.5 μg/kg and 0.5 μg/kg/h (group D_3_), and normal saline loaded with 50 ml/h for 10 min and maintained with 10 ml/h (group C). The loading dose of dexmedetomidine was infused intravenously at a constant speed with an infusion pump for 10 min.

### Study procedure

No patient received preoperative medication. All patients were monitored routinely with electrocardiogram (ECG), non-invasive blood pressure (NBP) and pulse oxygen saturation (S_P_O_2_) after entering the operating theater. Venous access was established in a wrist cephalic vein with a 20-G intravenous cannula (B. Braun, Melsungen, Germany). Ringer’s solution was transfused at a rate of 10–15 ml/kg/h. For those with normal Allen’s test results, the left radial artery was punctured under ultrasound guidance after local anesthesia to monitor the invasive blood pressure (IBP). IBP and cardiac function monitoring equipment (Most-care, Projecta Engineering Co., Ltd, Italy) was connected to the arterial line. Dexmedetomidine or normal saline loading dose was initiated after 5 min’s rest. In each group, the loading dose was infused intravenously for 10 min before induction of anesthesia, and then the maintenance dose was initiated. An anesthetist took charge of the preparation of dexmedetomidine and saline, which were covered with a towel when on the infusion pump. Another anesthetist who was blinded to the drugs and group allocations was responsible for data collection and intraoperative management. The patients and surgeons were both blinded to group allocations. All research data were recorded at 8.00 a.m. - 12.00 a.m.

Total intravenous general anesthesia was induced with midazolam 0.05 mg/kg, propofol 1.0–2.0 mg/kg, sufentanil 0.2–0.4 μg/kg and rocuronium 0.6 mg/kg. After sufficient muscle relaxation, the tracheal tube was inserted and fixed and then mechanical ventilation was performed. The ventilator parameters were set at inhaled oxygen concentration 60%, tidal volume 6–8 ml/kg, respiratory rate 12–14 times/min, inspiration/expiration ratio 1:2, positive end-expiratory pressure 3 cmH_2_O, and end-tidal carbon dioxide pressure 35–45 mmHg (1 mmHg = 0.133 kPa). General anesthesia was maintained with intravenous propofol 4–12 mg/kg/h, remifentanil 5–15 μg/kg/h, and dexmedetomidine/normal saline with the corresponding doses in each group. Muscle relaxation was maintained with intermittent intravenous injection of rocuronium. The depth of anesthesia was monitored with Narcotrend monitoring (MT Monitortechnik GmbH&Co.KG, Germany), the stage of which was maintained between D2 to E1 (sedation index 20 – 46) in all groups. Propofol and remifentanil were infused until surgery ending, and dexmedetomidine/saline and rocuronium stopped infusion at about 30 min before the end of surgery. Propofol and remifentanil doses were adjusted according to anesthesia depth from the Narcotrend monitoring. If mean arterial pressure (MAP) decreased > 20% of baseline value, ephedrine or phenylephrine was used. If HR decreased > 20% of basic value, atropine was injected. Cases were excluded if vasoactive drugs or atropine was used. Patients were called their names every 1 min until eyes opening after surgery finish. The tracheal tube was removed when the patient met extubation indications. All subjects were then transferred to the post-anesthesia care unit (PACU).

Cardiac circulation efficiency (CCE), maximum pressure gradient (dp/dt), HR, MAP, and systemic vascular resistance (SVR) were recorded at the following time points: just before dexmedetomidine infusion (T_1_), dexmedetomidine loading dose finish (T_2_), surgery beginning (T_3_), 30 min after surgery beginning (T_4_), 1 h after surgery beginning (T_5_), surgery ending (T_6_) and 1 h after transferring to PACU (T_7_). The twelve-lead ECG was measured at T_1_, T_2_, T_6_, T_7_, 24 h (T_8_), 48 h (T_9_), 72 h (T_10_) and 1 month (T_11_) postoperatively using the digital electrocardiograph (aECG-12PWL, Xiamen Nalong Technology Co., Ltd, China) with ECG paper speeding at 25 mm/s and the gain at 10 mm/mv. Measurements and analyzation of the ECG were manipulated by an electrophysiologist who was blinded to group allocations. Each ECG in leads II and V5 was measured for 3 complete P-QRS-T cycles, and the averages were recorded respectively. The PR in lead II interval was measured, and the intervals of QRS, QT, QTc and Tp-e were measured in lead V5. The QT interval was measured from the start of QRS complex to the T wave ending, and the T wave ending was the intersection of the descending branch tangent and the baseline. If U waves appeared, the T wave ending was regard as the nadir of the curve between T and U waves. QTc was calculated based on the Bazett formula [18], in which QTc = QT/$\sqrt[{}]{RR}$. The Tp-e was measured from the peak to the T wave ending. The index of cardiac electrophysiological balance (iCEB) was calculated according to iCEB = QT/QRS. Electrolyte concentrations were tested at T_1_, T_2_, T_5_, T_6_ and T_7_, and changes in potassium (K^+^) and ionized calcium (iCa^2+^) levels were recorded. The surgery time, duration of anesthesia (the time from dexmedetomidine or normal saline loading dose infusion to tracheal tube removal) and waking time from surgery were recorded, respectively. The dosages of propofol and remifentanil, total fluid intake from entering the operating theater to PACU and use of vasoactive drugs were recorded. The occurrence of arrhythmia during perioperative period was also recorded.

### Statistical analysis

PASS 15.0 (NCSS Llc., USA) was applied to sample size calculation in this research. QTc was the main research indicator, showing a mean ± standard deviation (SD) of 1.0 ± 12.2 ms after dexmedetomidine loading dose used in our pilot study. As a result, at least 15 patients should be included in every group to reach a power of 80% (β = 0.2) with two-tailed significance level of less than 0.05. An additional 20% was added to make up loss by protocol violations, 18 patients were recruited per group to minimize the impact of missing data consequently.

SPSS 23.0 (IBM Corporation, USA) was used for complete statistical analysis. Data with normal distributions were expressed as means ± SD. One-way analysis of variance (ANOVA) was used for comparisons between groups (variance homogeneity), and Welch’s ANOVA was used for variance nonhomogeneity. Repeated measurement ANOVA was used for repeated measurements data in groups, and categorical data were compared by chi-square (*χ*^*2*^) test or Fisher's exact test. Bonferroni correction was used for group differences between baseline and each time point. Two-sided *P* values of less than 0.05 were considered to be significant.

## Results

**Fig. 1:**
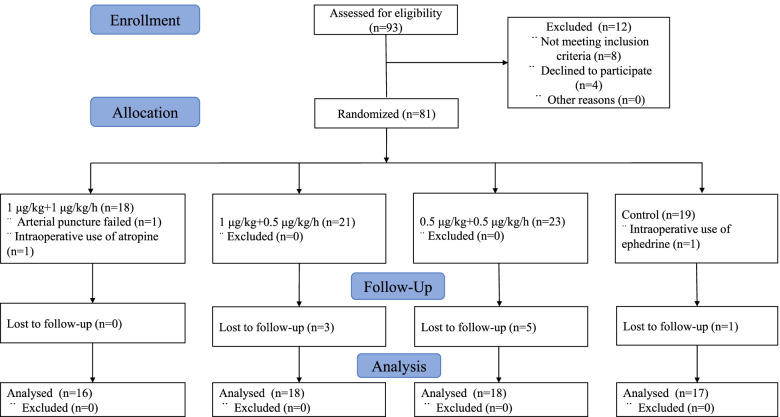
CONSORT flow diagram of enrollment

Of 93 patients originally recruited, 12 patients were excluded for refusal and failure to meet inclusion criteria. Therefore, 81 subjects were allocated randomly in this study, and data of 69 (33 males and 36 females) were available for the analysis finally. Figure 1 presents participants inclusion, reasons for exclusion, allocations and study procedures. The demographic characteristics of all subjects were presented in Table 1, and there were no significant differences among the four groups (*P* > 0.05). No significant differences were observed in clinical characteristics including surgery time, duration of anesthesia, waking time from surgery, total fluid intake from the operating theater to the PACU or patients with hypertension (*P* = 0.710, *P* = 0.462, *P* = 0.123, *P* = 0.631, *P* = 0.708, respectively) (Table 2). Compared with group C, the dosages of propofol and remifentanil in groups D_1_, D_2_ and D_3_ decreased significantly (*P* = 0.042, *P* < 0.001, respectively) (Table 2). No significant differences in changes of K^+^ and iCa^2+^ concentrations existed in any group (*P* > 0.05) (Table 3).

**Table 1:** Patients’ demographic data

	Group D_1_	Group D_2_	Group D_3_	Group C	*P* value
Gender (Male/Female)	8/8	9/9	8/10	8/9	0.985
Age (years)	49.1 ± 9.6	49.4 ± 11.2	43.1 ± 10.3	47.2 ± 9.9	0.237
Weight (kg)	64.5 ± 10.9	67.3 ± 7.6	65.4 ± 6.3	68.2 ± 8.1	0.553
Height (cm)	165.2 ± 8.2	166.8 ± 7.8	166.2 ± 7.3	166.2 ± 8.8	0.951
BMI (kg/m^2^)	23.6 ± 2.7	24.2 ± 1.9	23.7 ± 1.9	24.7 ± 2.3	0.452
ASA status I/II	9/7	9/9	13/5	11/6	0.549

Data are expressed as numbers or mean ± SD

*BMI* body mass index, *ASA* American Society of Anesthesiologists

**Table 2:** Clinical characteristics in the four groups during perioperative period

	Group D_1_	Group D_2_	Group D_3_	Group C	*P* value
Duration of Surgery (min)	100.6 ± 30.5	91.1 ± 35.7	88.6 ± 25.8	93.5 ± 31.6	0.710
Duration of anesthesia (min)	129.9 ± 33.0	115.3 ± 38.8	113.3 ± 26.2	118.1 ± 30.6	0.462
Waking time from surgery (min)	10.9 ± 3.5	9.7 ± 3.1	9.3 ± 2.5	8.4 ± 2.9	0.123
Total fluid intake from entering the operating theater to PACU (ml)	1171.9 ± 414.3	1180.6 ± 394.8	1030.0 ± 297.9	1152.4 ± 438.3	0.631
Patients with hypertension, n(%)	4 (25.0%)	2 (11.1%)	3 (16.7%)	2 (11.8%)	0.708
Dosage of propofol (mg/kg/h)	5.1 ± 1.0^△^	5.2 ± 1.0^△^	5.5 ± 1.1	6.1 ± 1.4	0.042
Dosage of remifentanil (μg/kg/h)	6.5 ± 2.4^*^	5.8 ± 1.7^*^	6.3 ± 2.2^*^	8.4 ± 1.4	< 0.001

Data are expressed as numbers or mean ± SD

*PACU* Post-anesthesia care unit

^△^ *P* < 0.05, ^*^ *P* < 0.01, compared with group C

**Table 3:** Electrolyte concentrations of K^+^ and iCa^2+^ in the four groups

		GroupD_1_ (*n* = 16)	GroupD_2_ (*n* = 18)	GroupD_3_ (*n* = 18)	GroupC (*n* = 17)	*P*^a^	*P*^b^	*P*^c^	*P*^d^	*P*^e^	*P*^f^
K^+^ (mmol/L)	T_1_	3.73 ± 0.20	3.72 ± 0.23	3.68 ± 0.18	3.80 ± 0.19	0.968	0.547	0.286	0.562	0.255	0.090
	T_2_	3.78 ± 0.19	3.76 ± 0.21	3.74 ± 0.20	3.82 ± 0.21	0.778	0.606	0.615	0.810	0.422	0.299
	T_5_	3.83 ± 0.21	3.79 ± 0.23	3.71 ± 0.18	3.81 ± 0.20	0.669	0.098	0.855	0.202	0.805	0.134
	T_6_	3.85 ± 0.21	3.85 ± 0.23	3.72 ± 0.24	3.82 ± 0.29	> 0.99	0.120	0.759	0.109	0.752	0.204
	T_7_	3.88 ± 0.26	3.88 ± 0.30	3.83 ± 0.20	3.91 ± 0.25	0.969	0.587	0.733	0.604	0.696	0.368
	*P*_T1-2_	0.841	> 0.99	0.474	> 0.99						
	*P*_T1-5_	0.282	0.903	> 0.99	> 0.99						
	*P*_T1-6_	0.104	0.057	> 0.99	> 0.99						
	*P*_T1-7_	0.172	0.121	0.153	0.760						
	*P*_T2-5_	> 0.99	> 0.99	> 0.99	> 0.99						
	*P*_T2-6_	> 0.99	0.334	> 0.99	> 0.99						
	*P*_T2-7_	0.739	0.280	0.916	0.827						
iCa^2+^ (mmol/L)	T_1_	1.15 ± 0.07	1.14 ± 0.03	1.13 ± 0.06	1.14 ± 0.04	0.434	0.193	0.515	0.588	0.900	0.510
	T_2_	1.15 ± 0.06	1.14 ± 0.04	1.14 ± 0.06	1.14 ± 0.02	0.510	0.488	0.389	0.972	0.826	0.856
	T_5_	1.15 ± 0.05	1.13 ± 0.04	1.13 ± 0.06	1.12 ± 0.02	0.327	0.247	0.148	0.852	0.614	0.749
	T_6_	1.15 ± 0.04	1.14 ± 0.04	1.15 ± 0.06	1.12 ± 0.03	0.752	0.833	0.151	0.588	0.244	0.091
	T_7_	1.16 ± 0.05	1.15 ± 0.04	1.14 ± 0.06	1.13 ± 0.03	0.677	0.148	0.085	0.285	0.174	0.756
	*P*_T1-2_	> 0.99	> 0.99	0.201	> 0.99						
	*P*_T1-5_	> 0.99	> 0.99	> 0.99	0.747						
	*P*_T1-6_	> 0.99	> 0.99	0.306	0.573						
	*P*_T1-7_	> 0.99	0.828	> 0.99	> 0.99						
	*P*_T2-5_	> 0.99	> 0.99	> 0.99	0.878						
	*P*_T2-6_	> 0.99	> 0.99	> 0.99	0.435						
	*P*_T2-7_	> 0.99	> 0.99	> 0.99	> 0.99						

Data are expressed as mean ± SD

*T*_*1*_ before dexmedetomidine infusion, *T*_*2*_ dexmedetomidine loading dose finish, *T*_*5*_ 1 h after surgery beginning, *T*_*6*_ surgery ending, *T*_*7*_ 1 h after entering PACU

*P*_T1-2_, *P*_T1-5_, *P*_T1-6_, *P*_T1-7_ for comparisons between T_1_ and T_2_, T_5_, T_6_, T_7_; *P*_T2-5_, *P*_T2-6_, *P*_T2-7_ for comparisons between T_2_ and T_5_, T_6_, T_7_; *P*^a^, *P*^b^, *P*^c^, *P*^d^, *P*^e^ and *P*^f^ for comparisons between groups D_1_ and D_2_, groups D_1_ and D_3_, groups D_1_ and C, groups D_2_ and D_3_, groups D_2_ and C, and groups D_3_ and C, respectively

### PR data

PR intervals in all groups were normal at T_1_. Compared with T_1_, the PR intervals were prolonged significantly at T_2_, T_6_ and T_7_ in group D_1_ (T_2_: *P* < 0.001, T_6_: *P* < 0.001, T_7_: *P* = 0.009), at T_2_ in group D_2_ (*P* < 0.001), and prolonged at T_2_, T_6_, and T_7_ (T_2_: *P* = 0.007, T_6_: *P* = 0.007, T_7_: *P* = 0.014) but shortened significantly at T_10_ (*P* = 0.008) in group D_3_. Compared with T_2_, the PR intervals were shortened significantly at T_8-11_ in groups D_1_ and D_2_ (D_1_, D_2_: *P* < 0.001, respectively) and at T_9-11_ in group D_3_ (T_9_: *P* < 0.001, T_10_: *P* < 0.001, T_11_: *P* = 0.004).

Compared with group C, the PR intervals were prolonged significantly at T_2_ in groups D_1_ and D_2_ (*P* < 0.001). Compared with groups D_1_ and D_2_, the PR intervals were shortened significantly at T_2_ in group D_3_ (*P* = 0.032, *P* = 0.019, respectively).

### QRS data

There were no significant differences in QRS intervals within and among the four groups (*P* > 0.05).

### QTc data

QTc values in all groups were normal and no differences existed at T_1_. Compared with T_1_, QTc was prolonged significantly at T_2_, T_6_ and T_7_ in group D_1_ (*P* < 0.001), at T_2_ and T_6_ in group D_2_ (T_2_: *P* < 0.001, T_6_: *P* = 0.023), and at T_6-8_ in group C (*P* < 0.01), while shortened significantly at T_2_ and T_10_ in group D_3_ (T_2_: *P* = 0.039, T_10_: *P* = 0.005). Compared with T_2_, QTc decreased significantly at T_10_ and T_11_ in group D_1_ (T_10_: *P* = 0.019, T_11_: *P* = 0.001), at T_9-11_ in group D_2_ (T_9_: *P* = 0.010, T_10_: *P* = 0.001, T_11_: *P* = 0.019), while increased significantly at T_6_ in group D_3_ (*P* = 0.001) and at T_6-8_ in group C (T_6_: *P* < 0.001, T_7_: *P* = 0.014, T_8_: *P* = 0.025).

Compared with group C, QTc in groups D_1_ and D_2_ were significantly prolonged at T_2_ (*P* = 0.001, *P* = 0.003, respectively), while shortened significantly at T_7_ and T_8_ in group D_3_ (*P* = 0.040, *P* = 0.021, respectively). QTc in group D_3_ was shortened significantly at T_2_, T_6_, T_7_, T_9_ and T_10_ compared with group D_1_ (T_2_: *P* < 0.001, T_6_: *P* = 0.044, T_7_: *P* = 0.001, T_9_: *P* = 0.016, T_10_: *P* = 0.027), and shortened significantly at T_2_ compared with group D_2_ (*P* < 0.001).

### Tp-e data

Tp-e values in all groups were normal and no differences existed at T_1_ and T_2_. There were no statistical differences among the four groups both at T_1_ and T_2_ (*P* > 0.05).

Compared with group C, Tp-e at T_8_ in group D_1_ was prolonged significantly (*P* = 0.007) while shortened significantly in group D_3_ (*P* = 0.032). Tp-e in group D_3_ was shortened significantly at T_7-9_ compared with group D_1_ (T_7_: *P* = 0.032, T_8_: *P* < 0.001, T_9_: *P* = 0.002) and at T_8_ and T_9_ compared with group D_2_ (T_8_: *P* < 0.001, T_9_: *P* = 0.007).

### iCEB data

iCEB values at T_1_ among the four groups were comparable. Compared with T_1_, iCEB at T_2_, T_6-8_ in groups D_1_ and D_2_ (D_1_: *P* < 0.001, D_2_: *P* < 0.001, *P* < 0.001, *P* = 0.022, *P* = 0.010, respectively), at T_6_ in group D_3_ (*P* < 0.001), and at T_6_ and T_7_ in group C (T_6_: *P* < 0.001, T_7_: *P* = 0.011) were significantly prolonged. Compared with T_2_, iCEB at T_10_ in groups D_1_ and D_2_ were significantly shortened (*P* = 0.008, *P* = 0.001, respectively), at T_6_ was prolonged and at T_8_ was shortened significantly in group D_3_ (T_6_: *P* = 0.038, T_8_: *P* = 0.031), and at T_6_ and T_7_ in group C was significantly shortened (T_6_: *P* < 0.001; T_7_: *P* = 0.044).

**Fig. 2:**
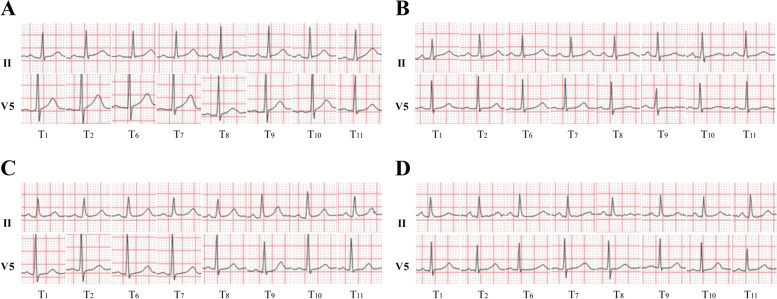
ECG at different time points in four groups during the perioperative period. **A** Changes of ECG in group D_1_. **B** Changes of ECG in group D_2_. **C** Changes of ECG in group D_3_. **D** Changes of ECG in group C. *II* Leads II of the 12-lead ECG; *V5* Leads V5 of the 12-lead ECG. T_1_: before infusion of dexmedetomidine; T_2_: dexmedetomidine loading dose finish; T_6_: surgery ending; T_7_: 1 h after transferring to PACU; T_8_: 24 h postoperatively; T_9_: 48 h postoperatively; T_10_: 72 h postoperatively; T_11_: 1 month postoperatively

iCEB at T_8_ in group D_3_ was significantly shortened compared with groups C, D_1_ and D_2_ (*P* = 0.047, *P* = 0.043, *P* = 0.024, respectively) (Table 4) (Fig. 2).

**Table 4:** Comparison of ECG indicators in the four groups

		GroupD_1_ (*n* = 16)	GroupD_2_ (*n* = 18)	GroupD_3_ (*n* = 18)	GroupC (*n* = 17)	*P*^a^	*P*^b^	*P*^c^	*P*^d^	*P*^e^	*P*^f^
PR (ms)	T_1_	147.4 ± 17.5	154.7 ± 15.6	149.2 ± 17.9	149.9 ± 17.9	0.223	0.763	0.678	0.342	0.415	0.903
	T_2_	170.6 ± 12.0	171.4 ± 14.5	158.0 ± 19.6	149.6 ± 19.2	0.878	0.032	0.001	0.019	< 0.001	0.141
	T_6_	164.6 ± 18.5	163.7 ± 13.4	161.4 ± 21.3	157.7 ± 20.3	0.888	0.621	0.290	0.715	0.343	0.555
	T_7_	161.6 ± 18.6	165.9 ± 17.4	162.1 ± 23.2	156.4 ± 18.1	0.515	0.942	0.446	0.552	0.151	0.390
	T_8_	154.9 ± 13.5	150.0 ± 16.1	152.7 ± 19.9	157.9 ± 16.3	0.393	0.701	0.608	0.627	0.165	0.359
	T_9_	147.1 ± 18.9	150.0 ± 13.9	143.8 ± 21.1	155.1 ± 19.5	0.646	0.614	0.220	0.322	0.423	0.078
	T_10_	146.1 ± 17.1	148.2 ± 14.1	141.6 ± 15.7	150.9 ± 19.5	0.707	0.439	0.403	0.238	0.631	0.102
	T_11_	145.8 ± 16.6	148.7 ± 15.6	145.6 ± 17.1	150.9 ± 17.3	0.619	0.964	0.385	0.577	0.695	0.348
	*P*_T1-2_	< 0.001	< 0.001	0.007	> 0.99						
	*P*_T1-6_	< 0.001	0.153	0.007	0.523						
	*P*_T1-7_	0.009	0.059	0.014	> 0.99						
	*P*_T1-8_	0.801	> 0.99	> 0.99	0.465						
	*P*_T1-9_	> 0.99	> 0.99	> 0.99	> 0.99						
	*P*_T1-10_	> 0.99	0.052	0.008	> 0.99						
	*P*_T1-11_	> 0.99	0.696	> 0.99	> 0.99						
	*P*_T2-6_	> 0.99	0.160	> 0.99	0.134						
	*P*_T2-7_	0.219	> 0.99	> 0.99	> 0.99						
	*P*_T2-8_	< 0.001	< 0.001	> 0.99	0.364						
	*P*_T2-9_	< 0.001	< 0.001	< 0.001	> 0.99						
	*P*_T2-10_	< 0.001	< 0.001	< 0.001	> 0.99						
	*P*_T2-11_	< 0.001	< 0.001	0.004	> 0.99						
QRS (ms)	T_1_	88.5 ± 7.9	88.6 ± 10.5	86.2 ± 9.8	84.0 ± 9.4	0.973	0.477	0.179	0.973	0.443	0.156
	T_2_	89.3 ± 10.6	87.7 ± 10.9	87.5 ± 12.1	84.2 ± 8.4	0.666	0.634	0.175	0.963	0.335	0.359
	T_6_	88.9 ± 9.7	87.3 ± 8.9	84.7 ± 10.7	84.5 ± 8.4	0.632	0.216	0.196	0.432	0.395	0.940
	T_7_	90.3 ± 9.6	88.7 ± 9.0	85.4 ± 9.5	85.1 ± 7.2	0.617	0.128	0.113	0.288	0.257	0.931
	T_8_	90.1 ± 10.5	88.4 ± 11.8	86.2 ± 9.2	85.4 ± 8.6	0.973	0.476	0.353	0.484	0.356	0.814
	T_9_	87.6 ± 7.7	87.7 ± 10.3	84.5 ± 9.2	84.6 ± 8.1	0.989	0.313	0.333	0.292	0.313	0.977
	T_10_	87.3 ± 8.5	87.7 ± 9.8	82.7 ± 7.9	86.6 ± 7.7	0.891	0.127	0.826	0.088	0.714	0.184
	T_11_	87.2 ± 6.6	85.9 ± 8.7	84.9 ± 9.8	85.8 ± 8.5	0.673	0.437	0.634	0.712	0.951	0.763
	*P*_T1-2_	> 0.99	> 0.99	> 0.99	> 0.99						
	*P*_T1-6_	> 0.99	> 0.99	> 0.99	> 0.99						
	*P*_T1-7_	> 0.99	> 0.99	> 0.99	> 0.99						
	*P*_T1-8_	> 0.99	> 0.99	> 0.99	> 0.99						
	*P*_T1-9_	> 0.99	> 0.99	> 0.99	> 0.99						
	*P*_T1-10_	> 0.99	> 0.99	0.830	> 0.99						
	*P*_T1-11_	> 0.99	> 0.99	> 0.99	> 0.99						
	*P*_T2-6_	> 0.99	> 0.99	0.247	> 0.99						
	*P*_T2-7_	> 0.99	> 0.99	> 0.99	> 0.99						
	*P*_T2-8_	> 0.99	> 0.99	> 0.99	> 0.99						
	*P*_T2-9_	> 0.99	> 0.99	> 0.99	> 0.99						
	*P*_T2-10_	> 0.99	> 0.99	0.061	> 0.99						
	*P*_T2-11_	> 0.99	0.804	> 0.99	> 0.99						
QTc (ms)	T_1_	413.8 ± 11.1	414.4 ± 12.2	416.2 ± 14.7	409.2 ± 15.5	0.882	0.605	0.341	0.703	0.259	0.134
	T_2_	427.0 ± 11.7	425.2 ± 12.6	408.1 ± 17.3	410.0 ± 14.9	0.719	< 0.001	0.001	0.001	0.003	0.690
	T_6_	429.9 ± 9.5	423.8 ± 10.1	419.6 ± 21.0	424.5 ± 14.3	0.228	0.044	0.292	0.389	0.888	0.323
	T_7_	428.7 ± 11.0	419.1 ± 13.9	413.3 ± 15.3	422.2 ± 9.0	0.031	0.001	0.148	0.171	0.468	0.040
	T_8_	419.2 ± 9.2	419.0 ± 13.4	413.0 ± 14.7	422.8 ± 10.6	0.965	0.147	0.398	0.148	0.361	0.021
	T_9_	418.0 ± 12.5	411.2 ± 16.2	405.7 ± 14.8	411.4 ± 13.9	0.174	0.016	0.192	0.258	0.970	0.250
	T_10_	413.8 ± 8.8	410.1 ± 12.7	404.0 ± 12.9	410.2 ± 15.0	0.397	0.027	0.419	0.151	0.977	0.149
	T_11_	408.1 ± 15.0	411.3 ± 13.3	412.1 ± 17.7	407.4 ± 16.5	0.554	0.457	0.897	0.874	0.464	0.375
	*P*_T1-2_	< 0.001	< 0.001	0.039	> 0.99						
	*P*_T1-6_	< 0.001	0.023	> 0.99	< 0.001						
	*P*_T1-7_	< 0.001	> 0.99	> 0.99	0.002						
	*P*_T1-8_	> 0.99	> 0.99	> 0.99	0.004						
	*P*_T1-9_	> 0.99	> 0.99	0.068	> 0.99						
	*P*_T1-10_	> 0.99	> 0.99	0.005	> 0.99						
	*P*_T1-11_	> 0.99	> 0.99	> 0.99	> 0.99						
	*P*_T2-6_	> 0.99	> 0.99	0.001	< 0.001						
	*P*_T2-7_	> 0.99	> 0.99	> 0.99	0.014						
	*P*_T2-8_	> 0.99	> 0.99	> 0.99	0.025						
	*P*_T2-9_	0.727	0.010	> 0.99	> 0.99						
	*P*_T2-10_	0.019	0.001	> 0.99	> 0.99						
	*P*_T2-11_	0.001	0.019	> 0.99	> 0.99						
Tp-e (ms)	T_1_	84.3 ± 13.2	81.7 ± 8.0	79.6 ± 8.8	82.5 ± 10.3	0.462	0.183	0.629	0.535	0.803	0.390
	T_2_	87.3 ± 14.7	82.1 ± 6.9	82.2 ± 7.7	81.9 ± 10.5	0.149	0.158	0.141	0.974	0.961	0.936
	T_6_	85.5 ± 9.6	83.8 ± 8.7	82.2 ± 9.1	85.9 ± 9.5	0.588	0.296	0.906	0.602	0.502	0.237
	T_7_	85.9 ± 8.7	83.4 ± 8.7	79.7 ± 8.0	84.2 ± 7.3	0.380	0.032	0.567	0.183	0.761	0.108
	T_8_	91.9 ± 11.0	87.4 ± 8.9	75.4 ± 10.0	82.5 ± 8.4	0.179	< 0.001	0.007	< 0.001	0.140	0.032
	T_9_	87.1 ± 11.2	85.3 ± 9.7	76.6 ± 8.8	81.2 ± 7.0	0.578	0.002	0.076	0.007	0.203	0.146
	T_10_	83.1 ± 10.8	82.6 ± 5.9	77.5 ± 7.5	81.9 ± 7.2	0.853	0.056	0.671	0.061	0.803	0.108
	T_11_	81.9 ± 9.2	78.9 ± 8.8	81.8 ± 10.1	80.5 ± 8.2	0.344	0.975	0.673	0.345	0.596	0.687
	*P*_T1-2_	> 0.99	> 0.99	> 0.99	> 0.99						
	*P*_T1-6_	> 0.99	> 0.99	> 0.99	> 0.99						
	*P*_T1-7_	> 0.99	> 0.99	> 0.99	> 0.99						
	*P*_T1-8_	0.160	0.733	> 0.99	> 0.99						
	*P*_T1-9_	> 0.99	> 0.99	> 0.99	> 0.99						
	*P*_T1-10_	> 0.99	> 0.99	> 0.99	> 0.99						
	*P*_T1-11_	> 0.99	> 0.99	> 0.99	> 0.99						
	*P*_T2-6_	> 0.99	> 0.99	> 0.99	> 0.99						
	*P*_T2-7_	> 0.99	> 0.99	> 0.99	> 0.99						
	*P*_T2-8_	> 0.99	> 0.99	0.222	> 0.99						
	*P*_T2-9_	> 0.99	> 0.99	0.666	> 0.99						
	*P*_T2-10_	> 0.99	> 0.99	0.358	> 0.99						
	*P*_T2-11_	0.115	> 0.99	> 0.99	> 0.99						
iCEB	T_1_	4.40 ± 0.48	4.51 ± 0.62	4.69 ± 0.69	4.70 ± 0.58	0.599	0.172	0.164	0.383	0.365	0.964
	T_2_	4.85 ± 0.67	4.96 ± 0.69	4.83 ± 0.85	4.70 ± 0.57	0.666	0.936	0.537	0.598	0.284	0.579
	T_6_	4.92 ± 0.62	4.97 ± 0.55	5.13 ± 0.64	5.22 ± 0.55	0.807	0.312	0.149	0.428	0.214	0.641
	T_7_	4.81 ± 0.53	4.82 ± 0.51	4.96 ± 0.60	5.04 ± 0.38	0.962	0.426	0.219	0.440	0.223	0.643
	T_8_	4.75 ± 0.54	4.87 ± 0.68	4.48 ± 0.61	4.93 ± 0.46	0.980	0.047	0.807	0.043	0.782	0.024
	T_9_	4.59 ± 0.50	4.70 ± 0.64	4.58 ± 0.62	4.78 ± 0.59	0.587	0.983	0.364	0.560	0.701	0.339
	T_10_	4.47 ± 0.54	4.51 ± 0.53	4.73 ± 0.57	4.48 ± 0.44	0.819	0.160	0.988	0.224	0.829	0.158
	T_11_	4.57 ± 0.39	4.69 ± 0.51	4.69 ± 0.60	4.60 ± 0.49	0.503	0.499	0.866	0.995	0.611	0.607
	*P*_T1-2_	< 0.001	< 0.001	0.876	> 0.99						
	*P*_T1-6_	< 0.001	< 0.001	< 0.001	< 0.001						
	*P*_T1-7_	0.001	0.022	0.095	0.011						
	*P*_T1-8_	< 0.001	0.010	0.968	0.624						
	*P*_T1-9_	0.989	> 0.99	> 0.99	> 0.99						
	*P*_T1-10_	> 0.99	> 0.99	> 0.99	0.875						
	*P*_T1-11_	> 0.99	0.849	> 0.99	> 0.99						
	*P*_T2-6_	> 0.99	> 0.99	0.038	< 0.001						
	*P*_T2-7_	> 0.99	> 0.99	> 0.99	0.044						
	*P*_T2-8_	> 0.99	> 0.99	0.031	0.947						
	*P*_T2-9_	0.317	0.253	0.319	> 0.99						
	*P*_T2-10_	0.008	0.001	> 0.99	0.647						
	*P*_T2-11_	0.331	0.304	> 0.99	> 0.99						

Data are expressed as mean ± SD

*ECG* Electrocardiogram, *QTc* Corrected QT, *Tp-e* Interval between the peak and the end of the electrocardiographic T wave, *iCEB* index of cardiac electrophysiological balance, *T*_*1*_ before infusion of dexmedetomidine, *T*_*2*_ dexmedetomidine loading dose finish, *T*_*6*_ surgery ending, *T*_*7*_ 1 h after transferring to PACU, *T*_*8*_ 24 h postoperatively, *T*_*9*_ 48 h postoperatively, *T*_*10*_ 72 h postoperatively, *T*_*11*_ 1 month postoperatively

*P*_T1-2_, *P*_T1-6_, *P*_T1-7,_ *P*_T1-8_, *P*_T1-9_, *P*_T1-10_, *P*_T1-11_ for comparisons between T_1_ and T_2_, T_6_, T_7_ T_8_, T_9_, T_10_, T_11_; *P*_T2-6_, *P*_T2-7_, *P*_T2-8_, *P*_T2-9_, *P*_T2-10_, *P*_T2-11_ for comparisons between T_2_ and T_6_, T_7_, T_8_, T_9_, T_10_, T_11_; *P*^a^, *P*^b^, *P*^c^, *P*^d^, *P*^e^ and *P*^f^ for comparisons between groups D_1_ and D_2_, groups D_1_ and D_3_, groups D_1_ and C, groups D_2_ and D_3_, groups D_2_ and C, and groups D_3_ and C, respectively

### Perioperative cardiac function and hemodynamic parameters

**Fig. 3:**
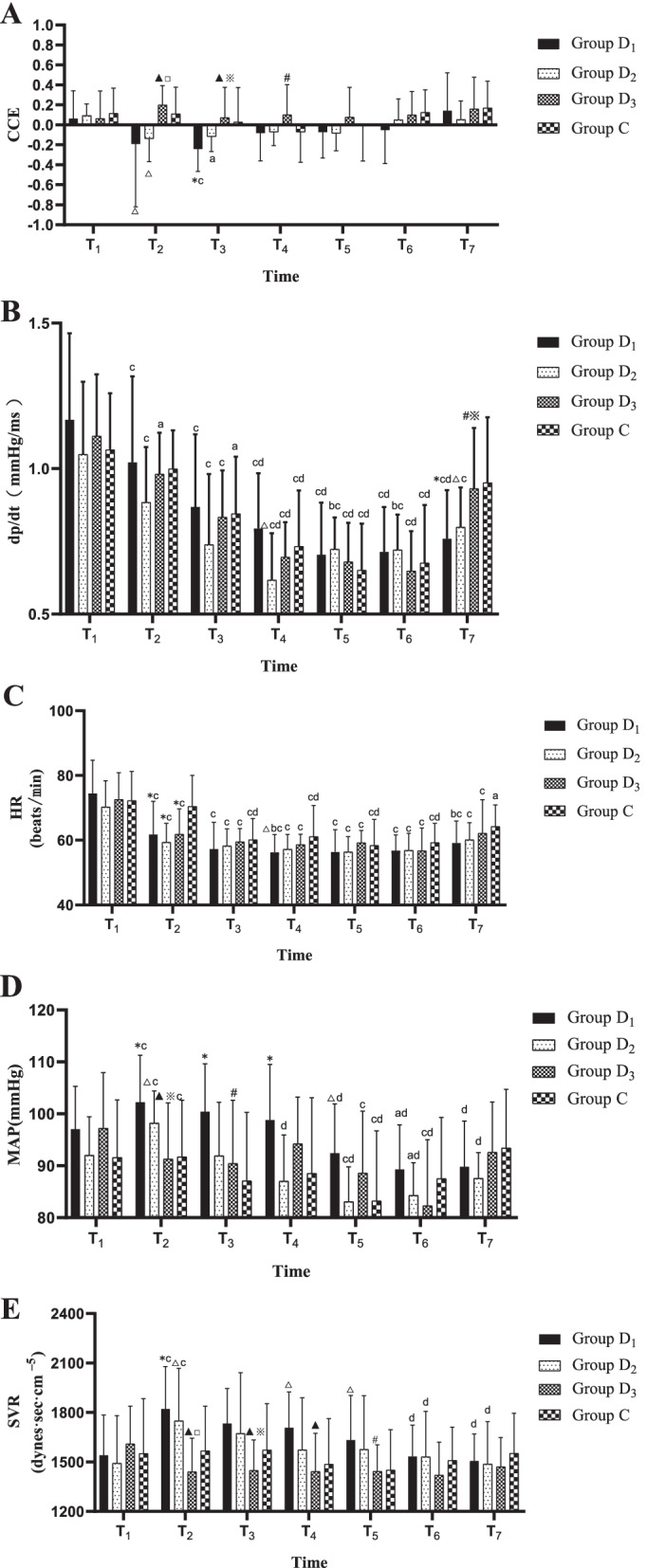
Cardiac function and hemodynamic indexes at different time points in the four groups. **A** Bar graphs of quantification of CCE change levels. **B** Bar graphs of quantification of dp/dt change levels. **C** Bar graphs of quantification of HR change levels. **D** Bar graphs of quantification of MAP change levels. **E** Bar graphs of quantification of SVR change levels. *CCE* cardiac circulation efficiency; *dp/dt* maximum pressure gradient; *HR* heart rate; *MAP* mean arterial blood pressure; *SVR* systemic vascular resistance. T_1_: before dexmedetomidine infusion; T_2_: dexmedetomidine loading dose finish; T_3_: surgery beginning; T_4_: 30 min after surgery beginning; T_5_: 1 h after surgery beginning; T_6_: surgery ending; T_7_: 1 h after transferring to PACU. ^a^ *P* < 0.05, ^c^ *P* < 0.01, compared with T_1_; ^b^ *P* < 0.05, ^d^*P* < 0.01, compared with T_2_; ^△^ *P* < 0.05, ^*^ *P* < 0.01, compared with group C; ^#^ *P* < 0.05, ^▲^ *P* < 0.01, compared with group D_1_; ^※^ *P* < 0.05, *P* < 0.01, Compared with group D_2_

Compared with T_1_, CCE decreased significantly in groups D_1_ and D_2_ at T_3_ (*P* = 0.001, *P* = 0.037). Compared with group C, CCE decreased significantly at T_2_ in groups D_1_ and D_2_ (*P* = 0.021, *P* = 0.047), and at T_3_ in group D_1_ (*P* = 0.005). Compared with group D_1_, CCE increased significantly in group D_3_ at T_2-4_ (T_2_: *P* = 0.003, T_3_: *P* = 0.001, T_4_: *P* = 0.046). Compared with group D_2_, CCE increased significantly in group D_3_ at T_2_ and T_3_ (T_2_: *P* = 0.007, T_3_: *P* = 0.037) (Fig. 3).

Compared with T_1_, dp/dt decreased significantly at T_2-7_ in groups D_1_ and D_2_, at T_2-6_ in group D_3_ (D_1_: *P* < 0.001, D_2_: *P* < 0.001, *P* < 0.001, *P* < 0.001, *P* < 0.001, *P* < 0.001, *P* = 0.001, D_3_: *P* = 0.010, *P* = 0.001, *P* < 0.001, *P* < 0.001, *P* < 0.001, respectively), and at T_3-6_ in group C (*P* = 0.020, *P* < 0.001, *P* < 0.001, *P* < 0.001). Compared with T_2_, dp/dt decreased significantly at T_4-7_ in group D_1_ (*P* < 0.001) and at T_4-6_ in groups D_2_, D_3_ and C (D_2_: *P* < 0.001, *P* = 0.013, *P* = 0.018, D_3_: *P* < 0.001, C: *P* < 0.001, respectively). Compared with group C, dp/dt decreased significantly in group D_1_ at T_7_ and in D_2_ at T_4_ and T_7_ (D_1_: *P* = 0.005, D_2_: *P* = 0.044, *P* = 0.020, respectively). Compared with groups D_1_ and D_2_, dp/dt increased significantly in group D_3_ at T_7_ (*P* = 0.010, *P* = 0.039) (Fig. 3).

Compared with T_1_, HR decreased significantly at T_2-7_ in each dexmedetomidine group (*P* < 0.001), and at T_3-7_ in group C (T_3-6_: *P* < 0.001; T_7_: *P* = 0.012). Compared with T_2_, HR decreased significantly at T_4_ in group D_1_ (*P* = 0.014), and at T_3-6_ in group C (*P* < 0.001). Compared with group C, HR decreased significantly at T_2_ in each dexmedetomidine group (D_1_: *P* = 0.005, D_2_: *P* = 0.001, D_3_: *P* = 0.004, respectively), and at T_4_ in group D_1_ (*P* = 0.013) (Fig. 3).

Compared with T_1_, MAP in group D_1_ increased at T_2_ (*P* = 0.003) and decreased at T_6_ (*P* = 0.024) significantly, increased at T_2_ (*P* < 0.001) and decreased at T_5-6_ in group D_2_ (T_5_: *P* = 0.002, T_6_: *P* = 0.012) significantly, decreased significantly at T_2_, T_5_ and T_6_ in group D_3_ (*P* < 0.001, *P* = 0.003, *P* < 0.001), and decreased significantly at T_5_ in group C (*P* = 0.006). Compared with T_2_, MAP in groups D_1_ at T_5-7_, D_2_ at T_4-7_, D_3_ at T_6_, and C at T_5_ decreased significantly (D_1_: *P* < 0.001, D_2_: *P* < 0.001, D_3_: *P* < 0.001, C: *P* = 0.005, respectively). Compared with group C, MAP in group D_1_ increased significantly at T_2-5_ (*P* = 0.002, *P* = 0.001, *P* = 0.009, *P* = 0.016), in group D_2_ increased significantly at T_2_ (*P* = 0.047). Compared with groups D_1_, MAP in group D_3_ decreased significantly at T_2_ and T_3_ (T_2_: *P* < 0.001, T_3_: *P* = 0.013). Compared with groups D_2_, MAP in group D_3_ decreased significantly at T_2_ (*P* = 0.033) (Fig. 3).

Compared with T_1_, SVR in groups D_1_ and D_2_ was significantly higher at T_2_ (*P* < 0.001, *P* < 0.001, respectively). Compared with T_2_, SVR in groups D_1_ and D_2_ was significantly lower at T_6_ and T_7_ (D_1_: *P* = 0.002, *P* < 0.001, D_2_: *P* = 0.031, *P* = 0.002, respectively). Compared with group C, SVR was significantly increased at T_2_, T_4_ and T_5_ in group D_1_ (T_2_: *P* = 0.008, T_4_: 0.019, T_5_: *P* = 0.048), and at T_2_ in group D_2_ (*P* = 0.047). Compared with group D_1_, SVR was significantly lower at T_2-5_ in group D_3_ (*P* < 0.001, *P* = 0.003, *P* = 0.005, *P* = 0.037). Compared with group D_2_, SVR in group D_3_ was significantly lower at T_2_ and T_3_ (T_2_: *P* < 0.001, T_3_: *P* = 0.016). No arrhythmia occurred during perioperative period in the four groups (Fig. 3).

## Discussion

Dexmedetomidine contains the imidazole ring, which has agonistic effects on α_2A_ and I_1_ imidazoline receptors [19], inhibits cardiac sympathetic nerve activity, and converts rapid ventricular and supraventricular arrhythmias [20]. It inhibits the activity of voltage-gated sodion (Na^+^) channel subtype α**—**Nav1.5 specifically expressed in cardiac tissues in a dose-dependent manner [21] to decrease the Na^+^ channel continuous current (I_Nap_) peak values under stress state, thus reduces effects on myocardial repolarization dispersion and the occurrence rates of malignant arrhythmia. Previous studies reported that dexmedetomidine could prolong the action potential duration of cardiomyocytes, reduce the incidence of early afterdepolarization, and decrease the autonomy of cardiomyocytes.

PR interval reflects AV conduction. In this study, the PR intervals in each dexmedetomidine group were significantly prolonged after loading dose, indicating that dexmedetomidine had inhibitory effects on AV conduction, which was consistent with the conclusion of Ergul et al.’s study [7]. The PR intervals in groups D_1_ and D_2_ were significantly prolonged than these in groups D_3_ and C when dexmedetomidine loading dose finish, and no significant difference existed between groups D_3_ and C. It indicated that the incidence of AV conduction block induced by dexmedetomidine might be related to its dose, that is, higher dose might produce higher incidence of AV conduction block. PR intervals recovered to normal levels at 48 h, 72 h and 1 month postoperatively in each dexmedetomidine group.

Abnormal prolongation of QTc usually indicates increased sensitivity to arrhythmia. In this study, the QTc intervals in groups D_1_ and D_2_ were significantly longer than the baseline values, while in group D_3_ shortened significantly after dexmedetomidine loading dose injection, suggesting that dexmedetomidine loading dose 1 μg/kg injected in 10 min could prolong ventricular repolarization duration and interfere with cardiac conduction system significantly, which was not conducive to cardiac electrophysiological stability. QTc in groups D_1_ and D_2_ was significantly prolonged than those in groups D_3_ and C when dexmedetomidine loading dose finish. Meanwhile, QTc in group D_3_ was significantly shorter than those in groups D_1_ and C at surgery ending, 1 h in PACU, 24 h and 48 h postoperatively. It indicated that dexmedetomidine in group D_3_ could reduce myocardial electrophysiological heterogeneity [22], suggesting that lower dose of dexmedetomidine could reduce the risk of myocardial electrical activity imbalance associated with increased sympathetic efferent stimulation during perioperative period [7, 23, 24]. Our research was different from Hammer et al.’s [8], which concluded that QTc was prolonged by dexmedetomidine, the reason might be related to the different inclusion criteria and medication scheme.

Tp-e is an indicator of the synchronization of myocardial cells repolarization and can be used as a predictor of torsades de pointes. Matthias et al. [25] found that dexmedetomidine loading doses of 0.25, 0.5, and 0.75 μg/kg injected in 1 min after general anesthesia induction had no significant influences on Tp-e intervals at 60 s after the injection. In our study, compared with group C, Tp-e at 24 h postoperatively in group D_1_ was significantly prolonged, while in group D_3_ shortened significantly, indicating an increased risk of desynchronization of myocardial cells repolarization existed in group D_1_, and suggesting that the effects of dexmedetomidine on ventricular transmembrane repolarization might be related to its dose. It has been reported that the Tp-e interval may be prolonged in response to sympathetic hyperactivity [26]. The shorter Tp-e interval in group D_3_ compared with group C in our study might be related to the balanced vagal efference and sympathetic activity originating from the lower dose of dexmedetomidine [27].

iCEB is an index that can reflect cardiac electrophysiological balance quickly and intuitively, which predicts the risk of arrhythmias, including torsades de pointes (TdP) and non-TdP ventricular tachycardia/ventricular fibrillation [28, 29]. The higher the iCEB values, the more likely imbalanced the electrophysiological homeostasis. In this study, iCEB in groups D_1_ and D_2_ increased significantly at dexmedetomidine loading dose finish, surgery ending and 24 h postoperatively compared with baseline values. Although iCEB in group D_3_ increased significantly compared with the baseline at surgery ending, it returned to the baseline at 24 h postoperatively and was significantly lower than groups D_1_, D_2_ and C, suggesting that higher dose of dexmedetomidine might not be beneficial to cardiac electrophysiological stability and lower dose of dexmedetomidine in D_3_ was more conducive to keep cardiac electrophysiological balance during perioperative period. Studies have shown [28] that abnormal iCEB elevations may be associated with excessive sympathetic activation and opening of cardiomyocyte cation channels, leading to drastic fluctuations in ion flow of action potential. The results of this study suggest that lower doses of dexmedetomidine are more advantageous than other doses applied.

In groups D_1_ and D_2_, SVR and MAP increased significantly compared to baseline values and were higher than groups C and D_3_ when dexmedetomidine loading doses completed, suggesting that dexmedetomidine at higher dose of 1 μg/kg infused for 10 min could stimulate the α_2B_ receptors on vascular smooth muscle cells and contract resistance blood vessels [30–32]. Meanwhile, dp/dt in groups D_1_ and D_2_ were significantly lower than basic values, which indicated that the cardiac ejection function was influenced to a certain extent.

CCE is the cardiac circulation efficiency, which reflects the work efficiency of the heart for maintaining the circulation dynamic balance. The higher the CCE, the greater the efficiency, which is negatively correlated with the sudden death rate [33]. In this study, CCE in groups D_1_ and D_2_ after dexmedetomidine loading dose were significantly lower than those in groups C and D_3_, indicating that dexmedetomidine loading dose 1 μg/kg might produce inhibitory effect on cardiac function. The reason might be related to the significantly increased SVR and cardiac afterload [34], producing a restrictive influence on cardiac function [35]. Dp/dt is the maximum pressure gradient, which is positively correlated with myocardial contraction function. In the current study, dp/dt in each dexmedetomidine group was significantly lower than the basic values after dexmedetomidine loading dose use, indicating that loading doses of 1 or 0.5 μg/kg could both inhibit myocardial contractility. While the dp/dt in group D_3_ was significantly higher than those in groups D_1_ and D_2_ at 1 h in PACU and had no significant difference compared with that in group C, illustrating that lower dose of dexmedetomidine at 0.5 μg/kg/h significantly improve the cardiac prognosis, which was consistent with the previous studies [36–38]. Hence, lower dose dexmedetomidine in D_3_ had no significant adverse effects on myocardial function during perioperative period.

In vitro studies [3, 39], dexmedetomidine reduced the I_Ca-L_ of cardiomyocytes significantly at a concentration of 10 ng/ml and above, that is, time of the ion channel inactivation was prolonged. In this study, HR in each dexmedetomidine group decreased significantly than the basic values and that in group C when the loading dose was completed. The reason might be related to the changes of Ca^2+^ ion channel inactivation [40]. Since catecholamines and cortisol had certain effects on cardiac electrophysiology, all research data in the current study were completed in the morning between 8 a.m. to 12 a.m. to avert the influences of day-night changes on cardiac electrophysiology [23]. Furthermore, basic values of K^+^ and iCa^2+^were comparable with standard values to avoid adverse effects on QTc, PR and QRS intervals [41].

Our study still has certain limitations. Considering that sevoflurane might prolong QTc intervals [42], this study only included patients under totally intravenous general anesthesia. The effects of dexmedetomidine on cardiac electrophysiology under combined intravenous and inhalational general anesthesia needs further study. Secondly, subjects with hypertension were not excluded in this study. Myocardial hypertrophy resulted from long-term hypertension might have certain impacts on myocardial repolarization and hemodynamics. Thirdly, we did not collect the intraoperative twelve-lead ECG limited by surgery manipulation. The effects of dexmedetomidine on electrocardia action and cardiac function still need to be proved by a multicenter and large-scale study.

## Conclusions

In summary, dexmedetomidine at a loading dose of 0.5 μg/kg and a maintenance dose of 0.5 μg/kg/h could maintain the stability of cardiac electrophysiology during perioperative period and has no significant adverse effects on CCE.

## Data Availability

The datasets used and/or analysed during the current study are available from the corresponding author on reasonable request.

## Abbreviations

ASA: American Society of Anesthesiologists
AV: Atrioventricular
BMI: Body mass index
CCE: Cardiac circulation efficiency
Dp/dt: Maximum pressure gradient
ECG: Electrocardiogram
HR: Heart rate
iCa^2+^: Ionized calcium
IBP: Invasive blood pressure
iCEB: Index of cardiac electrophysiological balance
K^+^: Potassium
MAP: Mean arterial pressure
Na^+^: Sodion
NBP: Non-invasive blood pressure
PACU: Post-anesthesia care unit
QTc: Corrected QT
S_P_O_2_: Pulse oxygen saturation
SD: Standard deviation
SVR: Systemic vascular resistance
